# Supplementary material for: A methodology for the design of experiments in computational intelligence with multiple regression models
Source: PeerJ. 2016 Dec 1;4:e2721. doi: 10.7717/peerj.2721 (PMC5136129; doi:10.7717/peerj.2721)
Supplement: Data S1 — Datailed results from UC Irvine Machine Learning Repository (Housing, Machine CPU, Wine Quality, Automobile and Parkinson) and the 3 Use Cases (Protein Corona, Gajewicz Metal Oxides and Aquatic Toxicity) [file peerj-04-2721-s001.zip › 6_UseCases/3_AquaticToxicity/RFRFE.details.csv.repeatedcv.split9.pdf]

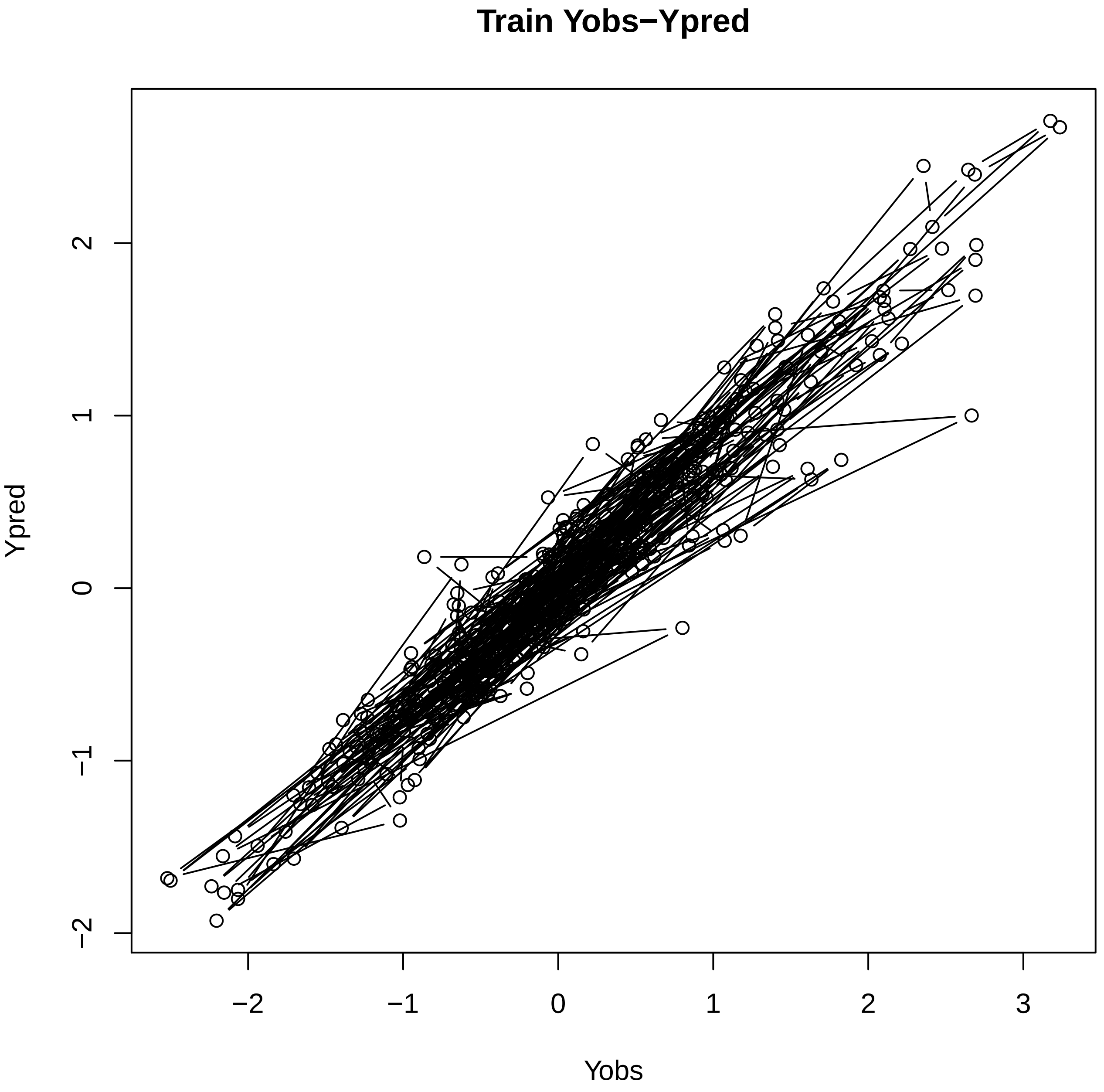

Train Yobs−Ypred
Ypred
Yobs
−2
−1
0
1
2
3

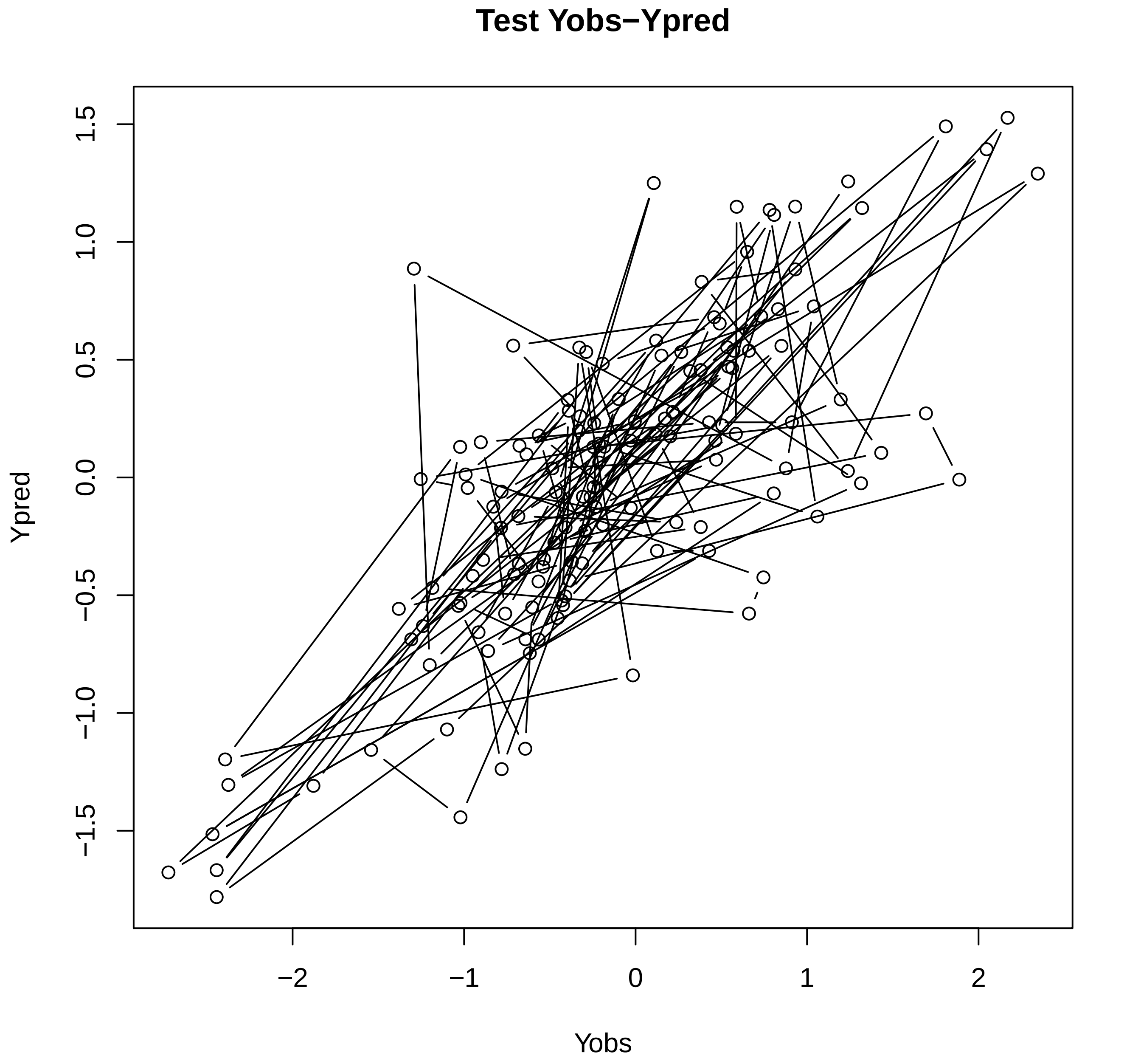
Test Yobs−Ypred
Ypred
1.5
1.0
0.5
0.0
−0.5
−1.0
−1.5
−2
−1
0
1
2
Yobs

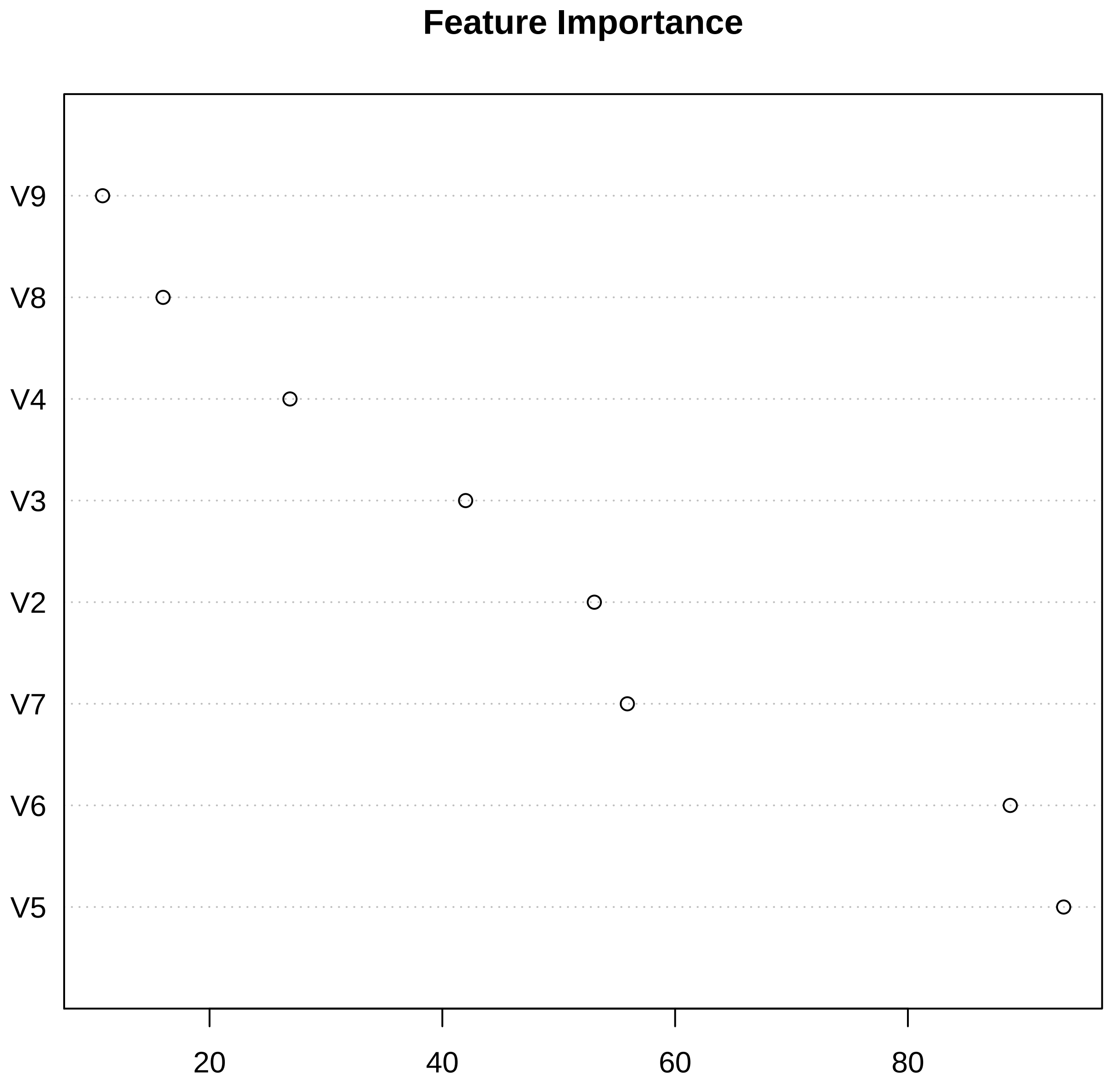
Feature Importance
V9
V8
V4
V3
V2
V7
V6
V5
20
40
60
80

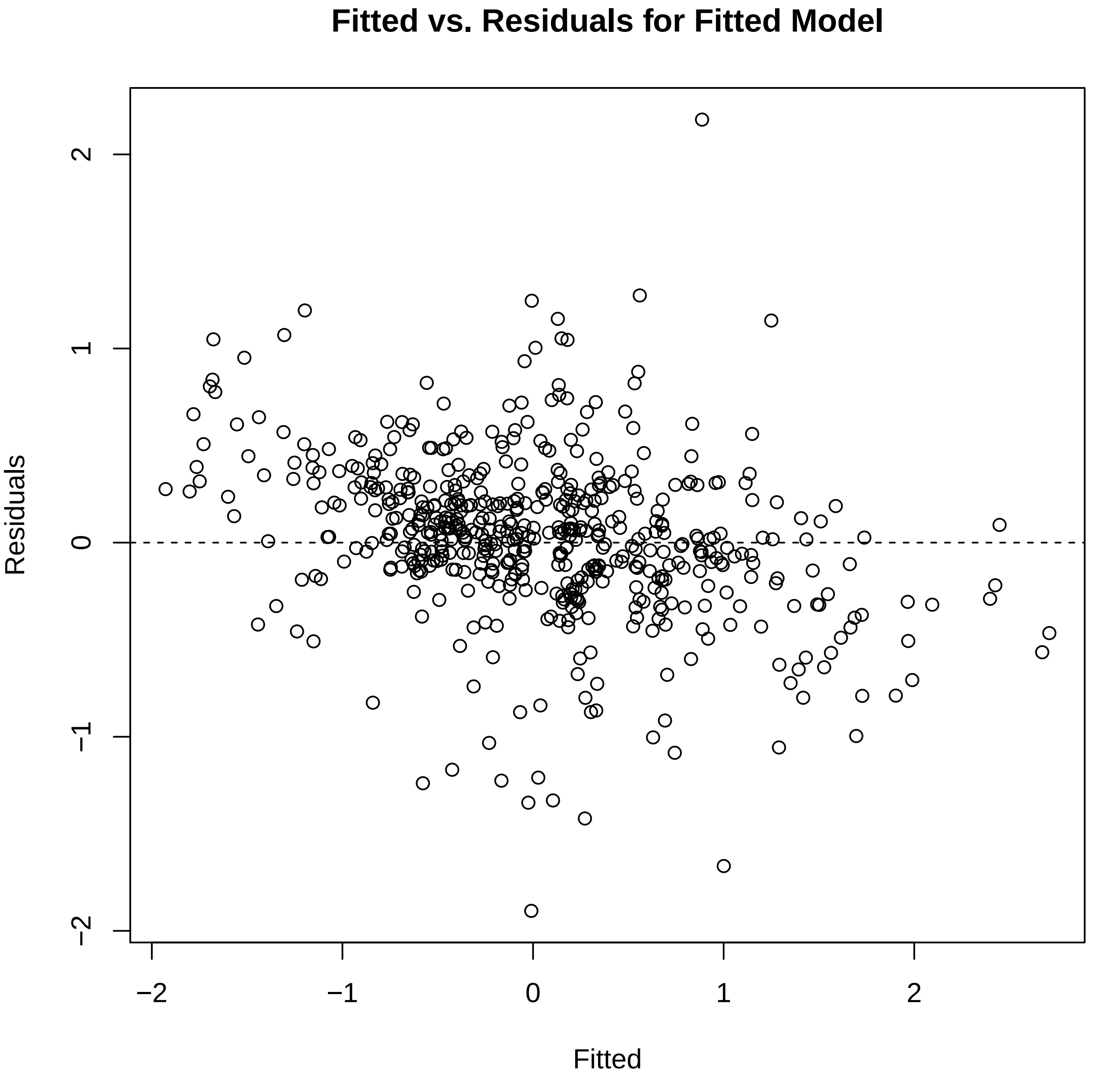

Fitted vs. Residuals for Fitted Model
Residuals
Fitted
2
1
0
−1
−2
−2
−1
0
1
2

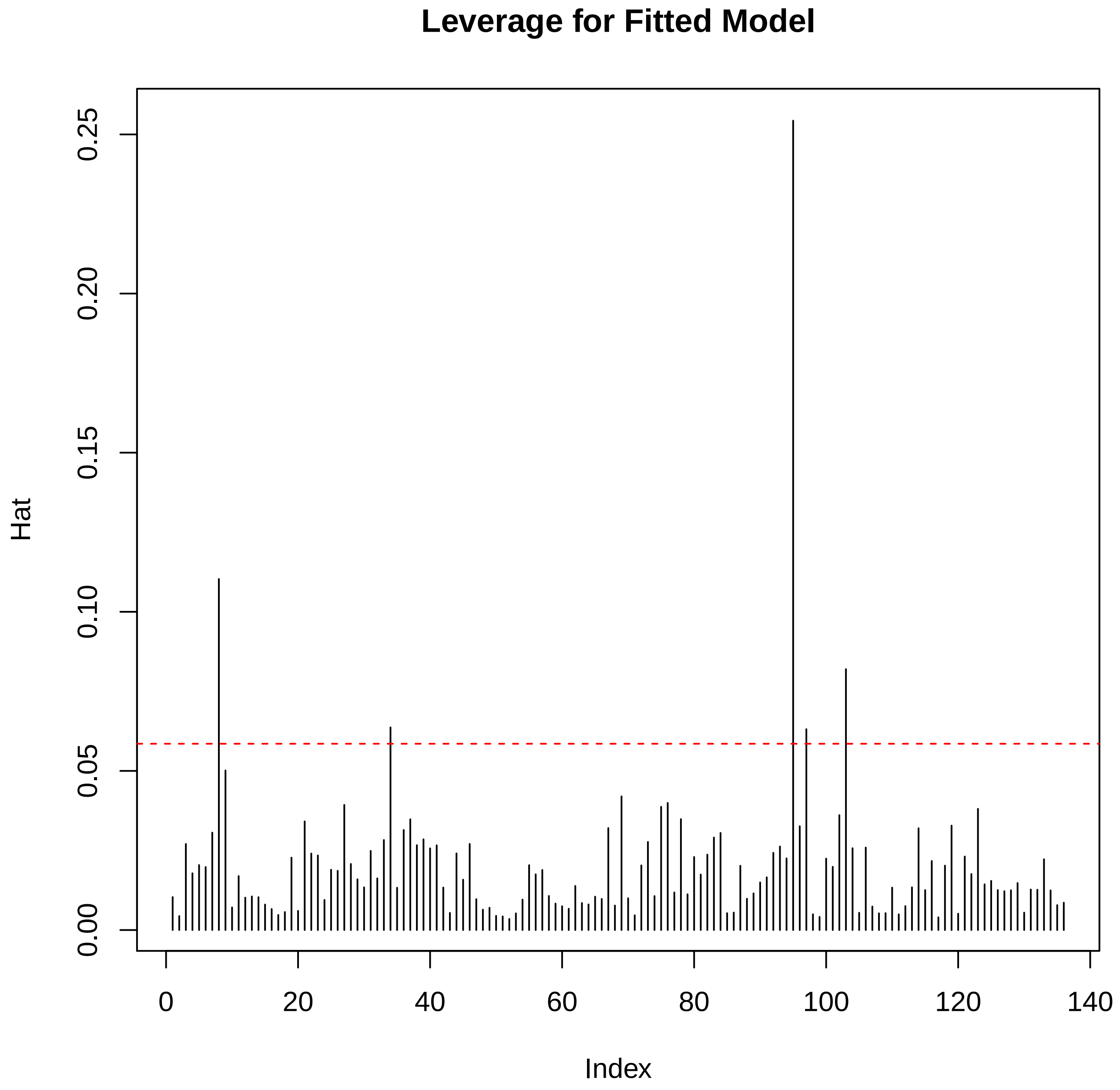

Leverage for Fitted Model
Hat
Index
0.00
0.05
0.10
0.15
0.20
0.25
0
20
40
60
80
100
120
140
